# Impact of 0.1% octenidine mouthwash on plaque re-growth in healthy adults: a multi-center phase 3 randomized clinical trial

**DOI:** 10.1007/s00784-021-03781-3

**Published:** 2021-01-22

**Authors:** Yvonne Jockel-Schneider, Ulrich Schlagenhauf, Hari Petsos, Stefan Rüttermann, Jana Schmidt, Dirk Ziebolz, Christian Wehner, Markus Laky, Thea Rott, Michael Noack, Barbara Noack, Katrin Lorenz

**Affiliations:** 1 Department of Periodontology, University Hospital Wuerzburg, Pleicherwall 2, 97070 Wuerzburg, Germany; 2 Department of Periodontology, Johann Wolfgang Goethe-Universität Frankfurt am Main, Theodor-Stern-Kai 7, 60596 Frankfurt am Main, Germany; 3 Department of Conservative Dentistry, Johann Wolfgang Goethe-Universität Frankfurt am Main, Theodor-Stern-Kai 7, 60596 Frankfurt am Main, Germany; 4 Department of Cariology, Endodontology and Periodontology, University of Leipzig, Liebigstraße 12, 04103 Leipzig, Germany; 5 Division of Conservative Dentistry and Periodontology, Medical University of Vienna, Sensengasse 2a, 1090 Wien, Austria; 6 Department of Operative Dentistry and Periodontology, University of Cologne, Kerpener Str. 32, 50931 Köln, Germany; 7 Department of Periodontology, Technische Universität Dresden, Fetscherstr. 74, 01307 Dresden, Germany

**Keywords:** Octenidine, Mouthrinse, Bacterial counts, Plaque index, Gingival index, Discoloration index

## Abstract

**Objectives:**

To investigate plaque inhibition of 0.1% octenidine mouthwash (OCT) vs. placebo over 5 days in the absence of mechanical plaque control.

**Materials and methods:**

For this randomized, placebo-controlled, double-blind, parallel group, multi-center phase 3 study, 201 healthy adults were recruited. After baseline recording of plaque index (PI) and gingival index (GI), collection of salivary samples, and dental prophylaxis, subjects were randomly assigned to OCT or placebo mouthwash in a 3:1 ratio. Rinsing was performed twice daily for 30 s. Colony forming units in saliva were determined before and after the first rinse. At day 5, PI, GI, and tooth discoloration index (DI) were assessed. Non-parametric van Elteren tests were applied with a significance level of *p* < 0.05.

**Results:**

Treatment with OCT inhibited plaque formation more than treatment with placebo (PI: 0.36 vs. 1.29; *p* < 0.0001). OCT reduced GI (0.04 vs. placebo 0.00; *p* = 0.003) and salivary bacterial counts (2.73 vs. placebo 0.24 lgCFU/ml; *p* < 0.0001). Tooth discoloration was slightly higher under OCT (DI: 0.25 vs. placebo 0.00; *p* = 0.0011). Mild tongue staining and dysgeusia occurred.

**Conclusions:**

OCT 0.1% mouthwash inhibits plaque formation over 5 days. It therefore can be recommended when regular oral hygiene is temporarily compromised.

**Clinical relevance:**

When individual plaque control is compromised, rinsing with octenidine mouthwash is recommended to maintain healthy oral conditions while side effects are limited.

**Supplementary Information:**

The online version contains supplementary material available at 10.1007/s00784-021-03781-3.

## Introduction

Antiseptic mouthwashes are commonly used in oral home care. They are recommended particularly in situations where the performance of efficacious mechanical plaque control is temporarily or permanently impaired, like in individuals in need of care, after oral surgical interventions, during orthodontic therapy with fixed appliances or as adjunct for gingivitis or periodontitis therapy [1–4]. Next to a mandatory very low systemic cytotoxicity, antiseptic agents suitable for use in a mouthwash need to have a long-lasting substantivity. This prevents an agent to be washed out of the mouth immediately by the salivary flow. In addition, a broad, unspecific antibacterial efficacy towards all bacterial species colonizing the oropharynx is essential [5–8].

Presently, chlorhexidine (CHX), a bisbiguanide antiseptic, comprehensively meeting all those requirements, is the most widely used antimicrobial agent in mouthwashes. Its clinical efficacy has been verified by a multitude of studies [9, 10].

More recently, octenidine dihydrochloride, a bispyridinamine, came into the focus of interest as another particularly suitable agent to be used in oral antiseptics due to properties rivaling those of CHX [11, 12]. It has an even lower systemic toxicity than CHX [13–15] possibly attributable to the lack of an amide and ester structure in its molecule [16]. Octenidine shows excellent and long-lasting adhesion to mucosal surfaces via its negative charge [17] and physically interacts with bacterial cell membrane components [18]. Its ability to attach to cells results in a residual depot effect on skin or wound tissue [19, 20]. Octenidine develops a broad antimicrobial activity, affecting Gram-positive and Gram-negative bacteria, chlamydiae, mycoplasmata, and fungi [12, 21, 22]. In clinical trials, octenidine showed promising results as a mouthwash agent regarding the reduction of bacteria and plaque inhibition [23].

### Aim

It was the aim of this clinical trial to evaluate the inhibitory effect of a 0.1% OCT mouthwash on plaque re-growth over 5 days in the absence of mechanical plaque control in healthy adult subjects, using a modified plaque re-growth model [24].

### Null hypothesis

No significant difference between both experimental groups regarding plaque re-growth assessed by plaque index (PI) [25] 5 days after initial professional mechanical plaque removal (PMPR) and the twice daily repeated application of the OCT mouthwash in comparison to the placebo mouthwash in the complete absence of personal mechanical plaque control.

## Material and methods

### Trial design

The investigation was designed as a prospective, randomized, placebo-controlled, double-blind, parallel group, multi-center phase 3 study, divided into two studies OML-III-A and OML-III-B. OML-III-A took place at the study centers of Dresden, Frankfurt, and Leipzig from January to December 2018. OML-III-B was performed at the study centers in Wuerzburg, Cologne, and Vienna from January to September 2018. The study was conducted in accordance with the principles of Good Clinical Practice (GCP) and the Declaration of Helsinki. It was approved by the ethics committees of the participating centers (study A: ethics committee of the University of Dresden EK 342082017, study B: ethics committee of the University of Wuerzburg 203/17_ff-me) and was registered at clinicaltrials.gov (study A: NCT03322124; study B NCT03378401) and the European Clinical Trials Database (study A: EudraCT No.: 2017-001697-42, study B: EudraCT No.: 2017-001698-18). All participants were informed about the study and signed the informed consent declaration. The studies were registered at clinicaltrials.gov: NCT03322124, NCT03378401.

The results presented in the following are based on the joint data set of the OML-III-A and OML-III-B studies, which followed identical study protocols and were performed concomitantly.

### Study population

Participants (male and female) were recruited among systemically healthy dental patients visiting the study centers in Dresden, Frankfurt, Cologne, Vienna, and Wuerzburg or among healthy volunteers from the resident population being asked for study participation by local advertisement.

Inclusion criteria were as follows: age ≥ 18 years; total mean gingival index GI ≤ 1.5 [26]; a minimum of 20 sound teeth including the Ramfjord teeth (16, 21, 24, 36, 41, 44) or their replacements (17, 11, 25, 37, 31, 45) [27, 28]; and 10 natural anterior teeth, necessary for the assessment of the discoloration index (excluding teeth restored with crowns or large vestibular fillings while teeth with only minor interdentally or orally located fillings were included).

Exclusion criteria were as follows: severe systemic diseases; necessity of antibiotic endocarditis prophylaxis; untreated caries with cavitation; presence of a GI score 3 at any tooth [26]; manifestation of periodontitis exceeding the presence of a Periodontal Screening and Recording Index (PSR) score 2 in more than two sextants or a PSR score > 3 in any sextant [29]; manifestation of other oral diseases including gingival overgrowth or mucosal diseases; orthodontic therapy; restoration with removable dentures; antibiotic therapy < 3 months prior to baseline examination; intake of systemically or locally acting corticosteroids (e.g., asthma sprays); xerostomia; hypersensitivity or allergy to the test product and its ingredients or to medications displaying a similar chemical structure; participation in another clinical study within the last 4 weeks before enrolment in and during this study; and pregnancy or breastfeeding.

### Composition and application of the experimental mouthwashes

The experimental mouthwashes were manufactured by NextPharma GmbH (Göttingen, Germany). They contained glycerol, sodium gluconate, citric acid, disodium phosphate dihydrate, macrogolglycerol hydroxystearate 40 EO, mint cool flavor PHL-167319, sucralose, and 0.1% octenidine (OCT mouthwash only) or phenoxyethanol 0.5% (placebo mouthwash only).

Study participants were instructed to rinse their mouth with 10 ml of the assigned mouthwash twice daily for 30 s each over a period of 5 days, resulting in a total of 10 applications. For dosing, a measuring cup was provided with the study drug package.

#### Study outcomes

##### Primary study outcome

Primary study outcome was the amount of plaque re-growth assessed by plaque index (PI) [25] 5 days after initial PMPR and the twice daily repeated application of the OCT mouthwash in comparison to the placebo mouthwash in the complete absence of personal mechanical plaque control. Following a modified plaque re-growth model (Addy et al., [24]), PI was recorded at screening (Vsc), baseline (V1), and at the final study visit (V2) on the Ramfjord teeth or their replacements at four sites per tooth (distovestibular, vestibular, mesiovestibular, and oral). The null hypothesis to be tested was that no significant difference regarding mean plaque index score existed between both experimental groups at V2.

##### Secondary study outcomes

Secondary study outcomes were the reduction of salivary bacterial counts after a single application of the experimental mouthwashes for 30 s at baseline and changes in mean GI scores [26] and DI scores [30] from baseline to the end of the study between OCT and placebo groups.

##### Assessment of salivary bacterial counts

For the assessment of salivary bacterial counts, the study participants were asked to rinse their mouth with 5 ml of sterile water for 30 s and to spit the originating saliva-water volume into a sterile screw-cap test tube. The samples were subsequently serially diluted, inoculated on Columbia blood agar plates + 5% sheep blood (bioMérieux SA, Marcy l’Etoile, France) and incubated at 37 °C for 48 h. The number of detected colony forming units (CFU) was used to calculate the number of CFU/ml for the respective dilution.

##### Assessment of gingival health

Gingival health was assessed by recording the gingival index [26] at Vsc, V1, and V2 on the Ramfjord teeth or their replacements at four sites per tooth (distovestibular, vestibular, oral, and mesiovestibular).

##### Tooth discoloration and questionnaire

The presence and extent of tooth discoloration were assessed at V1 after PMPR and after 5 days of rinsing (V2) at the vestibular sites of the anterior teeth according to the criteria of the discoloration index [30].

Furthermore, the study participants were instructed to document their daily consumption of staining beverages like coffee, tea, red wine, and juices, the use of chewing gum and menthol-containing lozenges, as well as tobacco smoking by a self-reported questionnaire.

##### Safety

At baseline and at the end of the study (day 5), the study participants were interviewed for the occurrence of related or unrelated adverse events.

##### Verification of application compliance

Application compliance was verified by instructing the study participants to return all empty, partially used, or unused containers of the experimental mouthwashes at the end of the study (V2). The volume of applied mouthwashes was calculated as difference in the weight of the assigned supply of mouthwash containers before handing out and after returning them to the study center.

##### Calibration, blinding and randomization

All clinical examiners of the participating study centers had been trained and calibrated prior to the onset of the study following established guidelines [31].

Investigators and other study personnel were blinded to the assignment of the study participants to the treatment groups throughout the study. Both experimental mouthwashes (OCT/placebo) were provided in bottles with identical packaging and labelling and had an identical appearance, color, and taste to ensure blinding of participants. The handing out to the study subjects in a 3:1 ratio (OCT vs. placebo) was performed by a study nurse not involved in the recording of the study data using a computer-generated randomization list. Randomization was stratified for GI baseline score (mean GI ≤ 1.0 vs. mean GI > 1.0).

##### Screening visit (Vsc)

At the screening visit, eligibility criteria were checked, and demographics, medical history, concomitant medication, and smoking habits were documented. PI [25] and GI [26] were recorded.

##### Visit 1 (V1, day 1, baseline)

**Fig. 1:**
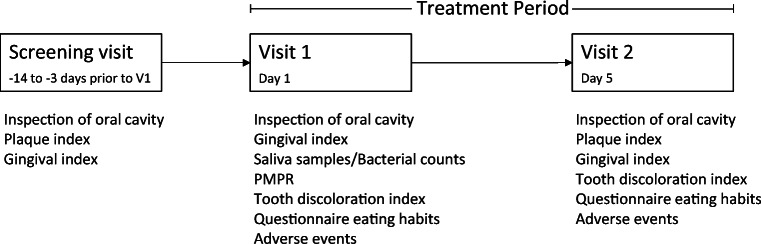
Study flow chart

At visit 1, eligibility criteria were re-checked, GI baseline scores were recorded, and a saliva sample was collected (Fig. 1). Subsequently the participants were randomly assigned to the OCT or the placebo group, performed a first supervised rinse with the assigned mouthwash for 30 s, followed 1 min later by the collection of another saliva sample. Subsequently all teeth were thoroughly cleaned from adhering bacterial biofilms, calculus, or superficial stains by PMPR using ultrasonic scalers and air-polishing devices.

The presence of non-removable tooth stains was documented using the DI, and participants were asked to perform a second supervised rinse. Instructions were given to repeat the rinsing twice daily at home for the next 4 days while refraining from any kind of mechanical plaque control and to perform the final rinse in the morning of day 5 within 4 h before the final examination. Finally, all participants received a supply of the assigned mouthwash sufficient for the next 5 days and a questionnaire for the self-reported documentation of consumed food and beverages during the observation period.

##### Visit 2 (V2, day 5, final examination)

At visit 2, the study participants returned the assigned mouthwash followed by the final assessment of GI, PI, and DI and the collection of completed questionnaires. A final PMPR concluded the study.

#### Statistical analysis

##### Sample size calculation

Sample size calculations performed for each study (OML-III-A/OML-III-B) revealed a number of 75 and 25 study subjects (OCT/placebo respectively) to be sufficient to detect a difference in mean PI of − 0.9 between the groups with a given power of *p* > 0.99, a given level of significance of *p* < 0.05, and an assumed mean PI score of 1.5 ± 0.6 for the placebo group.

##### Analysis of the primary outcome

Due to lack of normal distribution of data, the primary study outcome mean PI at day 5 of the trial was analyzed using van Elteren test (1-sided, *p* < 0.025). To assess the potential impact of co-variables, an additional analysis of co-variance (ANCOVA) with independent variables for treatment group, gingival status at V1 (factor for stratified randomization), total mean PI at screening, and study center was performed.

##### Analysis of secondary outcomes

Statistical analysis of the data of secondary study outcomes was performed by the van Elteren test using a 2-sided significance level of *p* < 0.05. The DI data were analyzed with both the van Elteren test and ANCOVA using a 2-sided significance level of *p* < 0.05.

## Results

### Recruitment, drop-outs, and protocol violations

**Fig. 2:**
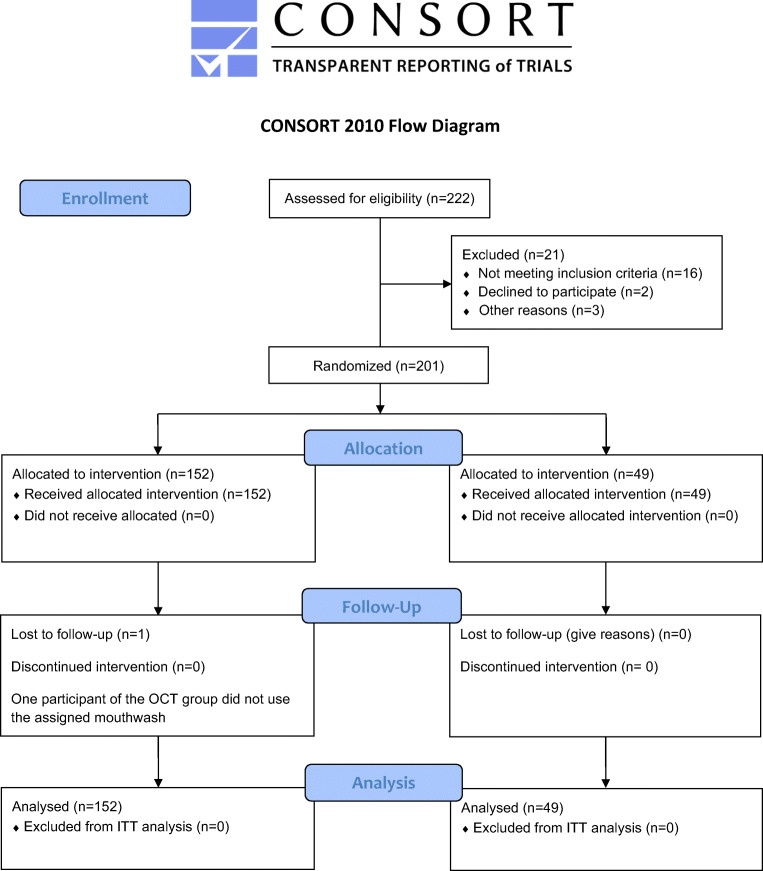
Consort 2010 flow diagram. Recruitment, drop-outs, and protocol violations during the study observation period

Recruitment and drop-outs are depicted in the CONSORT [32] flow diagram in Fig. 2. Two hundred one individuals were recruited and randomly assigned in a ratio of 3:1 to the OCT (*n* = 152) or the placebo (*n* = 49) group. Two hundred of them completed the study with a full data set. One participant of the OCT group did not use the assigned mouthwash. Because there were no statistically significant differences between the intention to treat and the per protocol analyses regarding any of the assessed variables, all results depicted in the following are based on the intention to treat analysis.

### Demographic data

The demographic data of the study participants are displayed in Table 1. Both treatment groups were balanced regarding gender (56% female/44% male), age (mean age: 26.2 years), and tobacco use. There were no significant differences between the groups regarding medical history and previous or current use of medication.

**Table 1:** Patient demographics

Demographics	All participants *n* = 201	OCT group *n* = 152	Placebo group *n* = 49
Age (years)—mean ± SD	26.7 ± 7.1	26.3 ± 6.6	25.3 ± 4.3
Female gender—*n* (%)	113 (56.2%)	85 (55.9%)	28 (57.1%)
Non-smokers—*n* (%)	151 (75.1%)	115 (75.7%)	36 (73.5%)

*SD* standard deviation, *n* numbers of subjects, intention to treat analysis set

### Plaque re-growth at day 5 (primary study outcome)

The results of the plaque re-growth analysis are depicted in Table 2. At V2, the observed median PI score of the OCT group (0.36, range 0.00–2.13) was significantly lower (*p* < 0.0001, van Elteren test) than the median PI score of the placebo group (1.29, range 0.04–2.01). Among all confounding variables tested, ANCOVA only revealed a center effect (*p* < 0.0001). The null hypothesis of no difference between both experimental groups at V2 therefore had to be rejected.

**Table 2:** Plaque index (PI), gingival index (GI), and discoloration index (DI)

	OCT (*n* = 152)				Placebo (*n* = 49)			
	Vsc	V1	V2	Change V1 to V2	Vsc	V1	V2	Change V1 to V2
PI								
Median Min-max	0.33 0.00–2.33	0.00 0.00–0.00	0.36 0.00–2.13	0.36* 0.00–2.13	0.38 0.00–1.83	0.00 0.00–0.00	1.29 0.04–2.01	1.29 0.04–2.01
GI								
Median Min-max	0.29 0.00–1.38	0.33 0.00–1.46	0.25 0.00–1.13	− 0.04^#^ − 1.04 to 0.75	0.29 0.00–1.25	0.29 0.00–1.04	0.38 0.00–1.08	0.00 − 0.54 to 0.58
DI								
Median Min-max	–	0.00 0.00–1.00	0.25 0.00–2.25	0.17^†^ − 0.16 to 2.25	–	0.00 0.00–0.58	0.00 0.00–1.08	0.00 0.00–1.08

*Between OCT and placebo groups; van Elteren test, 2-sided, *p* < 0.0001, intention to treat analysis set

#Between OCT and placebo groups; van Elteren test, 2-sided, *p* < 0.001, intention to treat analysis set

†Between OCT and placebo groups; van Elteren test, 2-sided, *p* < 0.0011 intention to treat analysis set

*OCT* octenidine mouthrinse, *Vsc* screening visit, *V1* visit 1, *V2* visit 2, *n* numbers of subjects, *max* maximum, *min* minimum

### Secondary study outcomes

#### Reduction of salivary bacterial counts

A single rinse with the OCT mouthwash reduced salivary bacterial counts significantly stronger than the placebo mouthwash. The observed median decrease in salivary bacterial counts was 2.73 lgCFU/ml for the OCT group vs. 0.24 lgCFU/ml for the placebo group (2-sided *p* < 0.0001; van Elteren test; Table 3).

**Table 3:** Bacterial counts in saliva

Bacterial counts	OCT (*n* = 152)			Placebo (*n* = 49)		
	Before 1st rinse	After 1st rinse	Count reduction (lgRF)	Before 1st rinse	After 1st rinse	Count reduction (lgRF)
Median Min-max (lgCFU/ml)	6.550 5.17–8.44	3.815 0.00–11.38	2.725* − 3.99 to 7.63	6.570 4.96–7.68	6.310 5.22–7.32	0.240 − 0.84 to 1.09

*Between OCT and placebo groups; van Elteren test, 2-sided, *p < 0.0001*, intention to treat analysis set

*OCT* octenidine mouthrinse, *CFU* colony forming units, *max* maximum, *min* minimum, *lgRF* log reduction factor, *n* number of subjects

#### Gingival index

The variation of recorded median GI scores within the observation period is displayed in Table 2. The median reduction of the recorded mean GI scores between OCT and placebo was statistically significant (2-sided *p* < 0.001, van Elteren test).

#### Discoloration of teeth

The median change in the DI scores from V1 to V2 was significantly more pronounced in the OCT group when compared to the placebo group (2-sided, *p* < 0.0011, van Elteren test, Table 2).

#### Eating and smoking habits

A greater proportion of subjects in the OCT than in the placebo group drank tea during the study (63.3% vs. 57.0%), but fewer subjects in this group smoked (25.8% vs. 32.7%) and consumed menthol dragées (13.9% vs. 20.4%). Other eating habits were similar between the two groups.

#### Adverse events

There were no severe treatment-emergent adverse events (TEAE). Overall, 63 subjects (31.5%) experienced mild (*n* = 59) or moderate (*n* = 4) TEAEs. In the OCT group, 53 out of 151 participants (35.1%) reported 71 TEAEs. In the placebo group, 10 out of 49 subjects (20.4%) reported the occurrence of 12 TEAEs. The most frequently reported TEAEs in the OCT group were dysgeusia (*n* = 32), tongue discoloration (*n* = 9), and headache (*n* = 4). In the placebo group, oral discomfort (*n* = 3), dysgeusia (*n* = 2), and headache (*n* = 2) occurred most often. The remaining events were reported by < 5% of individuals in any treatment group. Out of 83 TEAEs, 63 TEAEs were considered being treatment-related (possible, probably or definitely related), 56 of them were reported by OCT-group members and 7 by participants of the placebo group.

## Discussion

The analysis of the study data clearly demonstrated the superiority of the 0.1% OCT mouthwash over placebo in the inhibition of plaque re-growth over a 5-day period in the absence of mechanical plaque control. This evidence is in line with the observed marked reduction of salivary bacterial counts after a single rinse with the 0.1% OCT mouthwash. The validity of the plaque re-growth model by Addy and co-workers used in this trial for the evaluation of plaque re-growth inhibition [1, 24] is well established and has been successfully applied in many other clinical studies before [33–36].

The reduction of dental plaque formation over the period of 5 days was statistically significant and may be considered clinically relevant. In fact, subjects of the OCT group had a total median PI score of 0.36 after 5 days of OCT mouthwash use as the only personal oral hygiene measure compared to a median PI score of 1.29 in the placebo group. The median PI score in the OCT group at V2 roughly corresponded to the median PI score observed at V1 for the total of the study population (PI 0.33) supporting the conclusion that the regular use of the 0.1% OCT mouthwash has the same antibacterial efficacy as routine mechanical oral hygiene measures. Similar observations were made in other studies using mouthrinses containing 0.1% octenidine [23, 37–40] or other antiseptic agents (e.g., chlorhexidine) [33, 41, 42]. The distinct bacterial count reduction by the use of a 0.1% OCT mouthwash observed in this study confirms the data of the phase II trial [23].

In comparison to the golden standard CHX, bacterial reduction and plaque inhibition occurred to a similar extent [42–44]. So far, it can be speculated that OCT would have a similar effect on bacteria and plaque. However, a future clinical study with CHX as positive control should prove this hypothesis.

While the inclusion criteria allowed to include study subjects with mild to moderate chronic gingivitis up to a mean GI score of 1.5, the overall baseline mean GI score of the study population was GI 0.40. Only 13 recruited study participants (6%) had a mean GI score > 1.0 and were suffering from moderate gingivitis. Nevertheless, the use of the OCT mouthwash resulted in a statistically significant inhibition of gingival inflammation, although the observed median reduction of GI = 0.04 may not be considered clinically relevant.

As reported in many other studies evaluating antiseptic mouthrinses before, tooth and tongue discoloration was the most frequently reported adverse event followed by dysgeusia [23, 37]. The observed differences in DI between OCT and placebo were statistically significant but are considered clinically not relevant as they represent a median difference of 0.25 on a 0 to 3 grading scale only. As tea/coffee consumption, tobacco smoking and tobacco chewing are well known to have a significant impact on tooth discoloration [45], they were recorded during this study, but no effect was proven (ANCOVA, 2-sided, *p* > 0.05).

### Strengths and limitations

As the recording of PI and GI scores was restricted to the Ramfjord teeth, the true extent of plaque re-growth and accompanied gingival inflammation within the dentition might have been underestimated. However, this systematic bias would have affected both groups equally. The general validity of study data gained by the evaluation of the Ramfjord teeth has been verified in various other studies [28, 46, 47]. Correlations between the two approaches were high. Authors reported an underestimation of 0 scores and overestimation of 2 + 3 scores [48]. The pooled analysis of the studies increased the sample size and leads to more reliable results with increased precision and power as compared to the individual studies. A center effect was revealed for the primary parameter. Reasons for this could not be elucidated. However, if it was due to different investigator judgment, this subjective judgment was the same in test and control groups and therefore should not have had a negative impact on the outcome. A 3:1 ratio of participants in favor to the OCT group was chosen to increase the number of OCT subjects for safety evaluation of the experimental agent. This approach could have led to some overestimation of the effects.

## Conclusion

Repeated rinsing with a 0.1% octenidine mouthwash is an efficacious measure for the temporary inhibition of plaque re-growth and the maintenance of gingival health in the absence of personal mechanical plaque control during 5 days.

## Supplementary information

ESM 1 (DOC 218 kb)
